# Does the antisecretory peptide AF-16 reduce lung oedema in experimental ARDS?

**DOI:** 10.1080/03009734.2019.1685029

**Published:** 2019-11-08

**Authors:** Annelie Barrueta Tenhunen, Fabrizia Massaro, Hans Arne Hansson, Ricardo Feinstein, Anders Larsson, Anders Larsson, Gaetano Perchiazzi

**Affiliations:** a Hedenstierna Laboratory, Department of Surgical Sciences, Uppsala University, Uppsala, Sweden;; b Cardiac Anesthesia and Intensive Care, Anthea Hospital, GVM Care & Research, Bari, Italy;; c Institute of Biomedicine, University of Gothenburg, Göteborg, Sweden;; d Department of Pathology and Wildlife Diseases, National Veterinary Institute, Uppsala, Sweden;; e Department of Medical Sciences, Uppsala University, Uppsala, Sweden

**Keywords:** AF-16 antisecretory factor, ARDS, extravascular lung water, pulmonary oedema

## Abstract

**Background:** Acute respiratory distress syndrome (ARDS) is an acute inflammatory condition with pulmonary capillary leakage and lung oedema formation. There is currently no pharmacologic treatment for the condition. The antisecretory peptide AF-16 reduces oedema in experimental traumatic brain injury. In this study, we tested AF-16 in an experimental porcine model of ARDS.

**Methods:** Under surgical anaesthesia 12 piglets were subjected to lung lavage followed by 2 hours of injurious ventilation. Every hour for 4 hours, measurements of extravascular lung water (EVLW), mechanics of the respiratory system, and hemodynamics were obtained.

**Results:** There was a statistically significant (*p* = 0.006, two-way ANOVA) reduction of EVLW in the AF-16 group compared with controls. However, this was not mirrored in any improvement in the wet-to-dry ratio of lung tissue samples, histology, inflammatory markers, lung mechanics, or gas exchange.

**Conclusions:** This pilot study suggests that AF-16 might improve oedema resolution as indicated by a reduction in EVLW in experimental ARDS.

## Introduction

Acute respiratory distress syndrome (ARDS) is an inflammatory lung injury with acute onset, characterized by increased pulmonary vascular permeability, pulmonary oedema, increased lung weight, and loss of aerated lung tissue (1). Ashbaugh and colleagues described this syndrome in 1967 (2), and, despite reductions in both incidence and mortality (3), ARDS is still a significant health problem with high mortality (4). Survivors often have a reduced quality of life (5). Other than treatment of the underlying condition leading to ARDS, the therapy is mainly focussed on limiting ventilator-induced lung injury (VILI) and on adequate fluid management (3,6,7). Currently, there are no effective pharmacologic interventions specific for ARDS (6).

Antisecretory factor (AF) is an endogenous 41 kDa protein (8,9), detectable in most tissues and plasma (10,11). The protein has antisecretory and anti-inflammatory properties (11–13). The biologically active site of the protein resides in a 16-peptide fragment, AF-16, with the sequence (I)VCHSKTR (8,13). In experimental models, AF-16 reduces brain oedema (14–17) and interstitial fluid pressure in solid tumours, but does not affect healthy tissue (18). In clinical trials increased concentrations of AF in plasma is associated with a decrease of symptoms in different conditions such as inflammatory bowel disease, gastroenteritis, and Ménière’s disease (19–21). It is unknown whether AF or AF-16 has any effect on oedema resolution in the lungs. We hypothesised that the peptide AF-16 could reduce pulmonary oedema formation in a porcine model of ARDS by altering the quantity of extravascular lung water (EVLW), the inflammatory response, and the pressure–volume (PV) relation of the lung.

## Materials and methods

The study was approved by the Animal Ethics Committee in Uppsala (decision 5.8.18–01054/2017), and the care of the animals followed the National Institute of Health guide for the care and use of laboratory animals (NIH publications No 8023, revised 1978). The study was performed at the Hedenstierna Laboratory, Uppsala University, Sweden

### Anaesthesia and instrumentation

Twelve piglets (25–30 kg), of mixed Swedish, Hampshire, and Yorkshire breeds, were sedated with an intramuscular injection of Zoletil Forte (tiletamine and zolazepam) 6 mg/kg and Rompun (xylazine) 2.2 mg/kg. A peripheral intravenous catheter was inserted in an ear vein. After 5–10 min the animals were placed supine on a table and anaesthesia was induced with fentanyl 5–10 µg/kg i.v. and maintained with a continuous i.v. infusion of ketamine 30 mg/kg/h, midazolam 0.1–0.4 mg/kg/h, and fentanyl 4 µg/kg/h. After established anaesthesia, controlled by absence of reaction to painful stimulation between the front hooves, Esmeron (rocuronium) 25 mg/kg/h was added as muscle relaxant. During the first hour, 30 ml/kg/h of Ringer’s acetate was infused i.v. During the second hour, until established lung injury, Ringer’s acetate was infused at a rate of 20 ml/kg/h, followed by a maintenance infusion of 10 ml/kg/h during the rest of the protocol.

After induction of anaesthesia, the animals were tracheostomized, and an 8 mm internal diameter tube (Mallinckrodt Medical, Athlone, Ireland) was inserted in the trachea and connected to a ventilator (Servo I, Maquet, Solna, Sweden). Until the initiation of lung injury the lungs were ventilated with tidal volume (V_T_) 8 ml/kg, respiratory rate (RR) 30/min, inspiratory/expiratory time (I:E) 1:2, inspired oxygen concentration (F_I_O_2_) 0.7, and positive end-expiratory pressure (PEEP) 5 cmH_2_O.

An oesophageal catheter (Oesophageal catheter, Erich Jaeger GmbH, Höchberg, Germany) was positioned in the distal third of the oesophagus, and the correct position was assessed by a modified Baydur procedure by finding less than 10% difference between simultaneous measurement of the oesophageal and the occluded airway pressures during compression of the chest wall (22), in order to ascertain that the oesophageal measurements reflected the pressure changes at the pleural surface. The procedure consisted in temporarily occluding the airways by a clamp and externally compressing the chest wall while recording both airway and oesophageal pressure. The catheter provided continuous measurements of oesophageal pressure (P_ESO_). Airway pressure (P_AW_) and airway flow (V’_AW_) were measured at the airway opening during the entire protocol. Three different pressure transducers (DigimaClic Pressure Transducers, Special Instruments GmbH, Nördlingen, Germany) were used to measure P_AW_, P_ESO_, and gastric pressure (P_GA_), while V’_AW_ was acquired by a Fleisch pneumotachograph (Laminar Flow Element type PT, Special Instruments GmbH, Nördlingen, Germany) positioned between the endotracheal tube and the ventilator, connected to a differential pressure transducer (Diff-Cap Pressure Transducer, Special Instruments GmbH, Nördlingen, Germany).

A triple-lumen central venous catheter for fluid infusions and a pulmonary artery catheter (Edwards Life-Science, Irvine CA, USA) for measurement of cardiac output (CO) and pulmonary artery pressures were inserted via the right jugular vein. An arterial catheter was inserted in the right carotid artery for blood sampling and blood pressure measurement, and a pulse contour cardiac output (PiCCO) catheter (PV2015L20, Pulsion, Munich, Germany) was placed in the right femoral artery for estimation of EVLW evolution. Blood gases were analyzed on an ABL 3 analyzer (Radiometer, Copenhagen, Denmark) immediately after sampling, and venous admixture was calculated according to the shunt Equation (23). A midline mini-laparotomy was performed for catheterization of the urinary bladder for urine drainage.

### Study protocol

**Figure 1.**
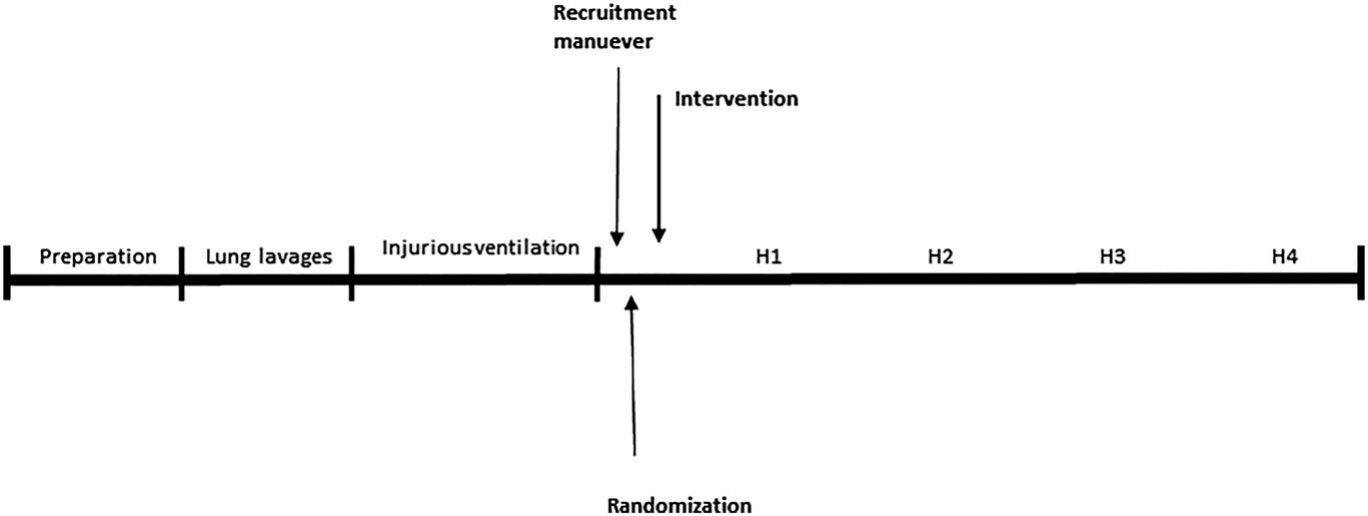
Experimental timeline. Lung lavages were repeated until the arterial oxygen tension/inspired oxygen tension (PaO_2_/F_I_O_2_) ratio was less than 27 kPa. Injurious ventilation consisting of plateau pressure of 36 cmH_2_O, fR 20/min, and inspiratory/expiratory time (I:E) 1:2, with zero end-expiratory pressures, was then initiated and maintained for 2 h. H1, H2, H3, H4 = measurements 1, 2, 3, and 4 h after intervention, respectively.

Preparation was followed by at least 30 min of stabilization, after which baseline measurements were performed (Figure 1). Lung injury was then induced using a two-hit injury model according to an established protocol (24). Lung lavages with 30 ml/kg of warmed isotonic saline were repeated until the arterial oxygen tension/inspired oxygen tension (PaO_2_/F_I_O_2_) ratio was less than 27 kPa. Injurious ventilation consisting of plateau pressure of 36 cmH_2_O, RR 20/min, and I:E 1:2, with zero end-expiratory pressures, was then initiated and maintained for 2 h. After the induction of lung injury, the animals were randomized to the intervention with AF-16 (*n* = 6) or to the control group (*n* = 6).

Directly after established lung injury the intervention group received AF-16 (sample No. 05501, KJ Ross Petersen ApS, Copenhagen, Denmark) 20 mg/kg in a solution of 50 mg/mL, administered as an infusion over 10 min in a central vein, while the control group received an equal amount of the vehicle (0.9% NaCl) at the same time point. A lung recruitment manoeuvre was performed for 2 min, by applying pressure-controlled ventilation; RR 6/min, PEEP 10 cmH_2_O, peak pressure of 40 cmH_2_O, and I:E 1:1. Thereafter, the following settings for mechanical ventilation were applied and maintained until the end of the protocol: volume-controlled ventilation, V_T_ 6 ml/kg, PEEP 14 cmH_2_O, F_I_O_2_ 0.7, and RR 40/min. This rate was chosen since it has previously been associated with formation of pulmonary oedema in this model (24).

At baseline, after establishment of lung injury and every hour during the following 4 hours’ duration of the protocol, EVLW, PaO_2_, PaCO_2_, pH, lactate, and base excess were measured. At the same time points, the main hemodynamic parameters (systemic and pulmonary pressures, CO, heart rate) were recorded. Pressure–volume (PV) curves of the respiratory system were obtained at baseline, directly after the induction of lung injury, and at 4 h after the intervention/vehicle, in steady-state conditions, just prior to euthanasia.

All respiratory signals were acquired by an analog-to-digital converter card (DAQ-card AI-16XE50, National Instruments Corp., Austin, TX, USA) controlled by the BioBench Software (ver. 1.0, National Instruments Corp., Austin, TX, USA), at a sampling frequency of 200 Hz. Inspiratory and expiratory airway volumes were obtained by integration of the airway flow (V’_AW_).

The PV relation was measured by delivering eight monotonically decreasing lung volumes, from P_AW_ 25 cmH_2_O to 0 cmH_2_O over PEEP. Each volume was delivered during steady-state ventilation, in volume control mode, and followed by an inspiratory hold manoeuvre (IHM) and an expiratory hold manoeuvre. Before the beginning of the decreasing ramp, in order to standardise the history of volume, we performed a recruitment manoeuvre applying a P_AW_ of 40 cmH_2_O for 40 s (25). Having the oesophageal catheter in place, we could measure the variation of transpulmonary pressure (P_TP_) as:

(1) $$\Delta P_{\text{TP}}=\left(P_{\text{AW},\text{plat}}-P_{\text{AW},\text{EE}}\right)-\left(P_{\text{ESO},\text{plat}}-P_{\text{ESO},\text{EE}}\right)$$

where P_AW,plat_ is airway pressure during IHM, P_ESO,plat_ is the oesophageal pressure at the same time, and P_AW,EE_ and P_ESO,EE_ are the corresponding airway and oesophageal pressures at the end of expiration (26). This way we could draw the PV curve of the lung in the different mentioned conditions.

The animals were euthanized with 100 mmol KCl i.v. at the end of the experiment under deep anaesthesia. The chest wall was then opened. Ventilation was maintained identical to the protocol in order to keep the pressure gradient between airway and vascular pressures during the sampling. The heart and the lungs were excised *en bloc*. Lung tissue samples were collected from both lungs from the following regions: apical-medial, medial-medial, caudal-dorsal, caudal-medial, and caudal-ventral. The samples were immediately immersed in 10% buffered formalin. A veterinary pathologist who was blinded to the experimental groups evaluated the samples histologically. In addition, samples from both lungs were analyzed for cytokines (TNF-α, IL-6) using an ELISA method. Wet-to-dry ratio was measured in the same lung regions from the right lung, and an average for each location was calculated. Samples were weighed, and dried in an oven, at 50 °C, until the weight did not differ between two measurements as described by Matute-Bello et al. (27).

### Statistics and data analysis

The number of animals to be included in this pilot study was determined according to the Mead resource Equation (28) that allows for determination of the sample size when the effect of a treatment is difficult to estimate *a priori*. We expressed values as means ± standard deviation (SD), or median and range where appropriate. The overall differences were analyzed by a two-way ANOVA using time and groups, and then the one-way ANOVA for repeated measurements (after established lung injury and every hour until the end of the protocol) to evaluate the within-group differences after starting treatment. The null hypothesis was that neither time nor treatment had an effect on the sampled data. The course of EVLW over time, in the treated versus non-treated group, was studied by applying a robust polynomial fitting (MatLab R2018, Curve Fitting Toolbox, MathWorks, Natick, USA) of the second degree, having a model of:

(2) $$y=p1*x^{2}+p2*x+p3$$

where *y* is the EVLW in mL and *x* is the time [hours]. The effect of ARDS induction was assessed by a paired *t* test. A difference of *p* < 0.05 was considered as statistically significant. Sigmaplot 12.5 (Systat Inc. Software, USA) was used for this statistical analysis.

In order to analyze and compare the PV curves from the different animals, the following procedure was applied. Each pressure value of the PV curves was divided by the maximum pressure that the same animal presented in healthy conditions at the highest volume. This indexing allowed for standardization of the comparison among different individuals and experimental phases while maintaining the morphology of the curve, as already published by Perchiazzi at al. for an analogous issue (29). The deflation limbs of all the PV curves were subjected to polynomial regressions of the second degree (MatLab R2018, Curve Fitting Toolbox, MathWorks, Natick, MA, USA), in order to verify whether they obeyed this model and in order to allow a further comparison between different individuals and phases of the experiments. The PV models were then compared using the *F* test (MatLab R2018, Statistics Toolbox, MathWorks, Natick, MA, USA), separately for treated and non-treated individuals in order to assess whether there was any statistically significant difference, firstly, between baseline conditions and post-lavage PV curves (hence attesting the validity of the lung injury model), and, secondly, between post-lavage PV curves and measurements taken after 4 h of treatment (hence attesting the presence of an effect after drug or vehicle administration).

## Results

No differences were found between the groups at baseline regarding hemodynamics, respiratory parameters, or body weight (Table 1). All animals survived the experiment until euthanasia.

**Table 1.** Measurements at baseline and at the end of the experiment. Values expressed as mean (SD). no statistically significant difference was found between the groups at baseline.

Parameters	AF-16		Control	
	Baseline	Final data	Baseline	Final data
Body weight (kg)	27.8 (1)	–	27.4 (1.7)	–
Arterial pH	7.50 (0.05)	7.32 (0.08)	7.51 (0.06)	7.33 (0.1)
PaCO_2_ (kPa)	5 (0.6)	7.5 (1.4)	5.2 (0.7)	7.9 (2.8)
PaO_2_ (kPa)	49.8 (4.6)	33.7 (10.7)	49.3 (2.1)	29.9 (12.9)
Peak pressure (cmH_2_O)	15 (1.8)	28.7 (2.9)	15.7 (1)	28 (2.3)
Plateau pressure (cmH_2_O)	11.2 (1.7)	19.5 (3)	11.5 (1.4)	19.8 (3.9)
Dynamic compliance (mL/cmH_2_O)	25.6 (4.2)	11.4 (2.4)	23.2 (3.6)	11.1 (2.1)
Mean arterial pressure (mmHg)	91 (5.6)	74 (17)	82 (10.7)	74 (13)
Cardiac output (L/min)	4 (0.6)	3 (0.7)	3.8 (0.5)	3.5 (1)
Extravascular lung water (mL)	324 (26)	425 (54)	331 (43)	462 (96)

PaCO_2_: arterial carbon dioxide tension; PaO_2_: arterial oxygen tension.

### Extravascular lung water

**Figure 2.**
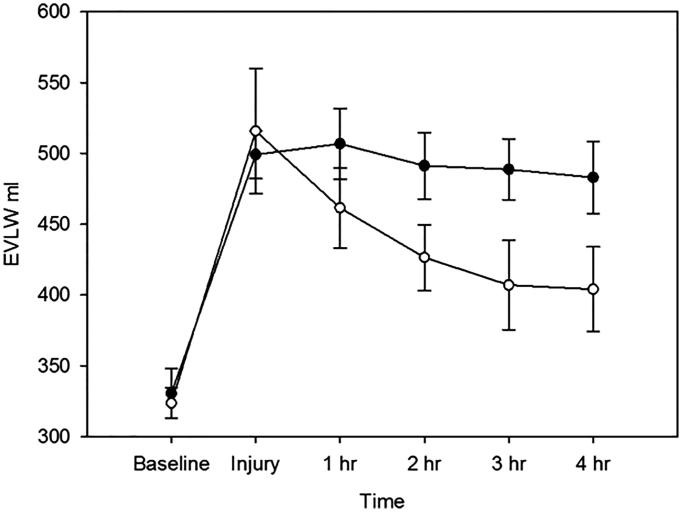
Extravascular lung water (EVLW) in mL at baseline, immediately after ventilator-induced lung injury and at 1, 2, 3, and 4 h post-damage. Black dots represent intervention group (AF-16), white dots represent control group.

After the induction of lung injury, the amount of EVLW increased (*p* < 0.001) in comparison to baseline. EVLW decreased over time in the intervention group as compared to the control group (*p* = 0.0057, difference of the means -51 ml, 95% confidence interval 86–15 ml) (Figure 2). In contrast, there were no statistically significant changes in the quantity of EVLW (*p* = 0.87) or by time (*p* = 0.12) in the control group after administration of the vehicle. The robust polynomial fitting yielded:

(3) $$\text{for}\;\text{the}\;\text{intervention}\;\text{group:}\;y=0.7*x^{2}-8.5*x+7.8\;\left(R^{2}=0.81\right)$$

(4) $$\text{for}\;\text{the}\;\text{controls:}\;y=-0.3*x^{2}+1.2*x-0.8\;\left(R^{2}=0.04\right)$$

### Wet-to-dry ratio

**Figure 3.**
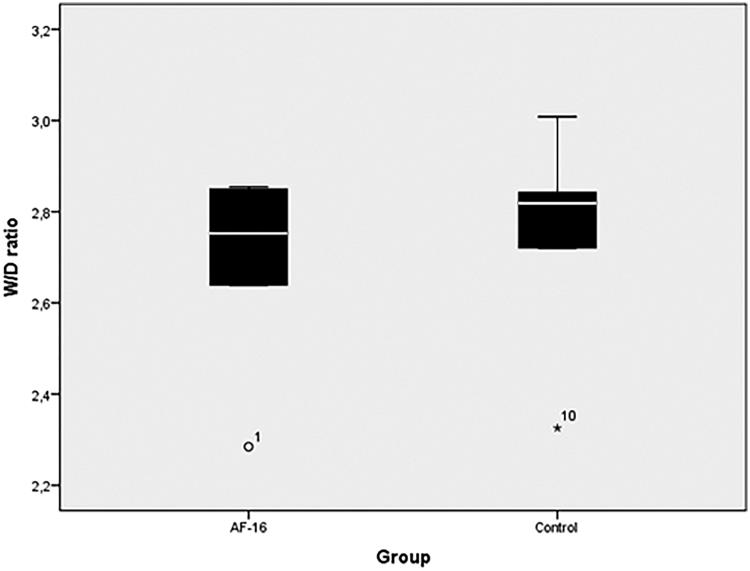
Wet-to-dry ratio, comparison between intervention (AF-16) and control group at the end of the experiment. Regional samples pooled from each animal. No statistically significant difference between groups.

There were no statistically significant differences in wet-to-dry ratio when pooling the regional samples from each animal (Figure 3) or when the different lung regions were analyzed separately.

### Lung mechanics

**Figure 4.**
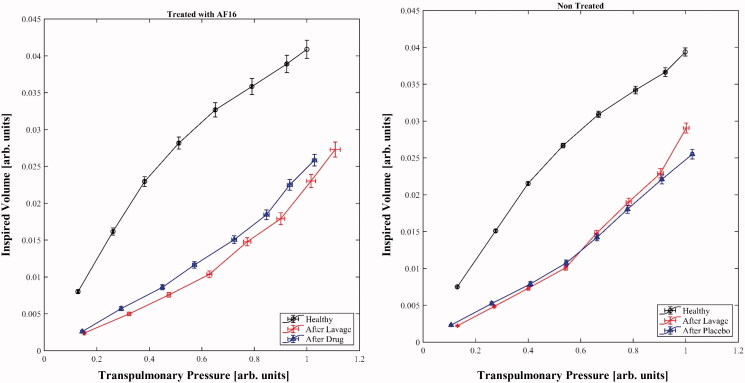
Pressure volume curves at airway opening; *x*-axes depict transpulmonary pressure (P_TP_), *y*-axes show inspired volume (V). Both axes are expressed in arbitrary units obtained by scaling both co-ordinates by the maximum value of pressure that the single animals have in healthy conditions. Using the same scaling factor, the morphology of the single curves remains unaffected.

The regression of the PV relations yielded statistically significant regression curves, including pooled data and data derived from treated and non-treated animals. The regressions separately subtending the three different phases of the experiment—healthy lungs, after induction of lung injury, and after administration of drug/vehicle—were statistically significant as well. Using the regression curves for estimating compliance at the same applied pressure in a standardized way, it is possible to infer that at 20 cmH_2_O compliance passes from 36.6 to 20 and then to 23.1 ml/cmH_2_O in the treated group, and from 39.9 to 22.6 and to 22.3 ml/cmH_2_O in the control group when considering the values sampled in the three different phases of the experiment. Using the *F* test to ascertain whether the curves displayed changes of their course, it is possible to note that in the case of the passage from healthy conditions to lung injury the PV curves were statistically different. The intervention with AF-16 did not change this course in a statistically significant way; the same happened in controls after the administration of vehicle (Figure 4).

### Gas exchange

After established lung injury the PaO_2_/F_I_O_2_ decreased from 70.8 (SD 4.9) to 12.6 (SD 4.4) kPa (*p* < 0.0001). No statistically significant difference in gas exchange could be distinguished between the intervention and control groups after start of administration (Table 2). Likewise, there was no difference in venous admixture or in pH between the two groups throughout the study.

**Table 2.** Gas-exchange data. After established lung injury the PaO_2_/F_I_O_2_ decreased from 70.8 kPa (SD 4.9) to 12.6 kPa (SD 4.4) (*p* < 0.0001), pooled data. No statistically significant difference could be distinguished in gas exchange between treated and non-treated groups after intervention. Values shown as mean (SD).

Group	Baseline	VILI	VILI + 1h	VILI + 2h	VILI + 3h	VILI + 4h
PO_2_ (kPa)						
AF-16	49.8 (4.6)	14.4 (4.4)	32.2 (15.6)	32.3 (10.1)	33.4 (10.2)	33.7 (10.7)
Control	49.3 (2.1)	10.8 (3.8)	27 (14)	30.6 (12.7)	32 (11.1)	29.9 (12.9)
PaO_2_/F_I_O_21_ (L/kPa)						
AF-16	71.1 (6.6)	14.4 (4.4)	46.1 (22.4)	46.1 (14.4)	47.7 (14.5)	48.1 (15.2)
Control	70.4 (3)	10.8 (3.8)	38.6 (20)	43.7 (18.1)	45.7 (15.9)	42.7 (18.5)
PCO_2_ (kPa)						
AF-16	5 (0.6)	4.3 (2.5)	7.6 (1.5)	7.6 (1.5)	7.5 (1.3)	7.5 (1.4)
Control	5.2 (0.7)	4 (1)	7.7 (2.1)	7.7 (2.6)	7.7 (2.7)	7.9 (2.8)
pH						
AF-16	7.5 (0.05)	7.55 (0.22)	7.29 (0.09)	7.3 (0.09)	7.31 (0.08)	7.32 (0.08)
Control	7.51 (0.06)	7.58 (0.09)	7.31 (0.09)	7.32 (0.1)	7.33 (0.1)	7.33 (0.1)
Lactate (mmol/L)						
AF-16	1.8 (0.48)	3.6 (0.9)	2.4 (1)	1.7 (0.5)	1.4 (0.3)	1.1 (0.2)
Control	1.5 (0.64)	2.8 (0.7)	1.5 (0.6)	1.2 (0.6)	1.1 (0.4)	1.1 (0.5)
Peak pressure (cmH_2_O)						
AF-16	15 (1.8)	42.2 (4.4)	28.8 (2.5)	29 (2.4)	28.3 (2.9)	28.7 (2.9)
Control	15.7 (1)	44 (4.2)	27.8 (1.6)	28.3 (2.1)	28.5 (2.6)	28 (2.3)
Plateau pressure (cmH_2_O)						
AF-16	11.2 (1.7)	31.2 (3.1)	18.7 (4)	19.5 (3.4)	17.7 (4.5)	19.5 (3)
Control	11.5 (1.4)	34 (2.3)	20 (3.5)	20 (3.7)	19.7 (4.1)	19.8 (3.9)
Driving pressure (cmH_2_O)						
AF-16	6.2 (1.7)	31.2 (3.1)	4.7 (4)	5.5 (3.4)	5.3 (3.3)	5.5 (3)
Control	6.5 (1.4)	34 (2.3)	7.7 (5)	6 (3.7)	5.7 (4.1)	5.8 (3.9)
Dynamic compliance (mL/cmH_2_O)						
AF-16	25.6 (4.2)	19.5 (5.6)	11.1 (2)	11 (2.3)	11.3 (2.3)	11.4 (2.4)
Control	23.2 (3.6)	20.5 (3.9)	11.6 (1.8)	11.4 (1.8)	11.1 (2)	11.1 (2.1)

PaCO_2_: arterial carbon dioxide tension; PaO_2_: arterial oxygen tension; PaO_2_/F_I_O_2_: arterial oxygen tension inspired oxygen tension; VILI: ventilator-induced lung injury.

### Cytokines

Lung homogenates from the two groups did not differ in IL-6 or TNF-α concentrations in a statistically significant way. The IL-6 concentration was 511 (SD 377) pg/mL and 551 (SD 472) pg/mL in the AF-16 group and the control group, respectively. The values for TNF-α were 132 (SD 72) pg/mL and 135 (SD 88) pg/mL.

### Histology

**Figure 5.**
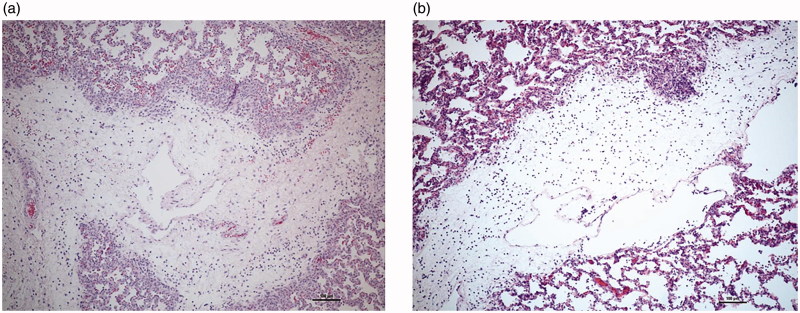
Representative lung tissue samples from an animal treated with AF-16 (a) and a control animal (b). Both images show prominent oedema in the interlobular septa with leukocyte infiltration and dilated lymphatic capillaries.

The histological analysis identified interstitial oedema, leucocyte infiltration, emphysema, and atelectasis to a varying extent (Figure 5). There was no significant difference between the two groups with respect to oedema or inflammatory activity (Supplementary Table 1, available online).

### Hemodynamics

Mean arterial pressure, CO, and heart rate decreased after established VILI with no significant difference between groups. Circulatory parameters were stable throughout the experiment in both groups (Supplementary Table 2, available online). Fluid administration was standardized according to the protocol, and both groups maintained an adequate diuresis throughout the experiment.

## Discussion

This pilot study in experimental ARDS suggests that the antisecretory peptide AF-16 might improve oedema resolution, indicated by a decrease in EVLW. At the same time, there were no effects on gas exchange, respiratory mechanics, inflammatory response, or alveolar damage.

We used a two-hit model for ARDS, consisting of lung lavage followed by injurious ventilation. The surfactant depletion by lung lavage primes the lung for injurious ventilation, and we established a ventilator-induced lung injury with a resultant mean PaO_2_/F_I_O_2_ of 12.6 (SD 4.4), which equals severe ARDS in humans, according to the Berlin definition (1). The model was stable as indicated by no change in EVLW, gas exchange, and lung mechanics over the time following the induction of injury, in the control group.

The ARDS model used in this protocol has several features similar to human ARDS, such as rapid onset, histological findings of tissue injury with evidence of an inflammatory response, increased pulmonary vascular permeability, and severe hypoxaemia, all being important features of acute lung injury in an animal model (27,30). Thus, the inflammatory, histological, and lung mechanical characteristics can be assumed to mirror ARDS in patients.

AF-16 has been found to reduce oedema formation in different conditions, e.g. brain oedema related to mild-to-moderate traumatic brain injury (14), and to reduce interstitial pressure in solid tumours (18). In the airways endogenous AF is localized to the epithelium of the trachea and the bronchial tree, as well as in mononuclear cells in the lamina propria (8). In the pulmonary acini AF is localized to type II cells and lung macrophages, the former localization indicating a possible regulatory role of AF in the secretion of pulmonary surfactant (8). AF also has neuromodulatory effects and inhibits chloride permeation over neuronal membranes (8,11,17,31–33). Our hypothesis was that administration of AF-16 would improve oedema resolution in this porcine ARDS model. Indeed, we found that EVLW decreased in the AF-16-treated group, and although there was a tendency to a reduced wet-to-dry ratio of the lung samples and a positive effect on lung mechanics, neither reached statistical significance.

We hypothesized that the significant reduction of EVLW of approximately 50 ml was too small to be detected in a statistically significant way in the small samples that were subjected to the drying process. On the other hand, it is worth mentioning that the assessment by the wet-to-dry ratio includes a blood component (which inevitably remains inside the sample) while EVLW does not. This last is ‘the amount of water that is contained in the lungs outside the pulmonary vasculature, that is, the sum of interstitial, alveolar, intracellular, and lymphatic fluids’ (34). Furthermore, we could not discern any positive effect on gas exchange. This might be explained by the longer time it takes for inflammatory compared with non-inflammatory oedema to be resorbed and thus to any improvement in respiratory compliance and oxygenation.

AF-16 downregulates the inflammatory response in several models (8,9,11,12). However, in this study there was no difference in cytokine levels or in histology between the controls and the AF-16 group. These results were indeed expected, since the observation period was short, and any immunological effect would first appear after a longer time. On the other hand, this study was not designed to primarily assess the immune response by AF-16, but mainly to assess the possible oedema resolution properties.

The study of PV curves was performed in order to observe any possible lung mechanical effect of the intervention. Notably, the induction of lung injury reduced the lung compliance, confirming the validity of the applied model. Dealing with different individuals and different experimental phases, we decided to standardize the curves dividing the pressure component by its maximum value measured in healthy condition. Lung compliance is also a function of body weight, and this procedure allowed this potential problem to be eliminated. A careful examination of the graph of the treated animals shows that the curves before and after the administration of AF-16 seem to follow different courses, rendering a better compliance after the treatment. Although this difference cannot be considered statistically different, the observation opens the question whether we could have had a better signal-to-noise ratio using other doses/timings of drug administration or simply by increasing the sample size.

This study has many limitations; first, it is an animal study with an artificial lung condition, where the surfactant depletion by lung lavage primes the lung for injurious ventilation. Human ARDS, in contrast, is often the result of complex interactions of disease (directly or indirectly affecting the lung), co-morbidities, and genetic predisposition (3,30). No animal model reproduces the full characteristics of human ARDS, and interspecies variability in lung injury mechanisms and response to intervention can by no means be ruled out (30). Furthermore, although AF-16 was given i.v. in a dose that was higher than in the experimental models of brain injury, the insult was also more severe, and we cannot disprove that the intervention with AF-16 would be more effective at an even higher dose. Moreover, the number of studied animals was limited, and this affected the magnitude of the treatment effects that could have been considered statistically significant: small variation between the groups might not have been detected. Finally, the intervention was not blinded to the investigators, since this could not be achieved for practical reasons. The analyzing pathologist, however, was blinded.

In summary, we found that EVLW was reduced by the antisecretory peptide AF-16, but we could not find any major effect on inflammation, gas exchange, or lung mechanics. Thus, further long-term experimental studies are necessary to assess whether AF-16 has any important effect on oedema resolution in ARDS and to pinpoint underlying mechanisms.
